# PNU-74654 Suppresses TNFR1/IKB Alpha/p65 Signaling and Induces Cell Death in Testicular Cancer

**DOI:** 10.3390/cimb44010016

**Published:** 2022-01-04

**Authors:** Wen-Jung Chen, Wen-Wei Sung, Chia-Ying Yu, Yu-Ze Luan, Ya-Chuan Chang, Sung-Lang Chen, Tsung-Hsien Lee

**Affiliations:** 1 Institute of Medicine, Chung Shan Medical University, Taichung 40201, Taiwan; mimic1024@gmail.com (W.-J.C.); flutewayne@gmail.com (W.-W.S.); 2 School of Medicine, Chung Shan Medical University, Taichung 40201, Taiwan; cyyu2015@gmail.com (C.-Y.Y.); vm6vul4@gmail.com (Y.-Z.L.); raptor7037@gmail.com (Y.-C.C.); 3 Department of Urology, Chung Shan Medical University Hospital, Taichung 40201, Taiwan; 4 Department of Obstetrics and Gynecology, Chung Shan Medical University Hospital, Taichung 40201, Taiwan; 5 Division of Infertility Clinic, Lee Women’s Hospital, Taichung 40201, Taiwan

**Keywords:** PNU-74654, TNF receptor-1, apoptosis, testicular cancer

## Abstract

Testicular cancer (TC) is a rare malignancy worldwide and is the most common malignancy in males aged 15–44 years. The Wnt/β-catenin signaling pathway mediates numerous essential cellular functions and has potentially important effects on tumorigenesis and cancer progression. The search for drugs to inhibit this pathway has identified a small molecule, PNU-74654, as an inhibitor of the β-catenin/TCF4 interaction. We evaluated the therapeutic role of PNU-74654 in two TC cell lines, NCCIT and NTERA2, by measuring cell viability, cell cycle transition and cell death. Potential pathways were evaluated by protein arrays and Western blots. PNU-74654 decreased cell viability and induced apoptosis of TC cells, with significant increases in the sub G1, Hoechst-stained, Annexin V-PI-positive rates. PNU-74654 treatment of both TC cell lines inhibited the TNFR1/IKB alpha/p65 pathway and the execution phase of apoptosis. Our findings demonstrate that PNU-74654 can induce apoptosis in TC cells through mechanisms involving the execution phase of apoptosis and inhibition of TNFR1/IKB alpha/p65 signaling. Therefore, small molecules such as PNU-74654 may identify potential new treatment strategies for TC.

## 1. Introduction

Testicular cancer is a rare malignancy in males worldwide and accounts for about 1% of newly diagnosed male cancers every year [1]. It also shows a specific age distribution, as it is most commonly encountered in males aged 15–44 years [2]. For unknown reasons, the incidence of this male cancer has been increasing in many developed countries in the last decades [3]. TC has a close relationship with cryptorchidism, but other known risk factors include prior TC, a family history of a father or brother with TC, increased adult height (per 5 cm increase), ethnicity and infertility [3,4]. Despite the increasing incidence, the overall five-year survival is as high as 97% with effective treatment [4]. However, the development of effective treatments depends on knowledge of the mechanisms that cause testicular tissues to become cancerous.

Current evidence now implicates the Wnt/β-catenin signaling pathway as part of the mechanism involved in the development of several malignancies. This pathway mediates numerous essential cellular functions, such as proliferation, differentiation, apoptosis and cell migration [5]. *INT1*, the first gene identified in the Wnt/β-catenin pathway, shows high similarity in both humans and mice; therefore, it is considered a highly conserved pathway evolutionarily among various species [6]. Wnt proteins are secreted extracellularly by cells and function through a receptor-mediated pathway [7]. By contrast, β-catenin is a component of the cell adhesion complex and plays an essential role in cell–cell adhesion [8]. In the canonical Wnt/β-catenin signaling pathway, WNT proteins are activated by binding to a transmembrane Frizzled family receptor and the LDL receptor-related protein (LRP) co-receptor and this inhibits the activity of glycogen synthase kinase-3β (GSK-3β). In this Wnt activation state, the β-catenin destruction complex is disrupted by activated Dishevelled protein (DVL) and the phosphorylation of β-catenin is prevented, thereby allowing β-catenin accumulation to occur within the cytoplasm. Then, β-catenin translocates into the nucleus, where it interacts with T-cell factor/lymphoid enhancer factor (TCF/Lef) to activate the TCF/Lef transcription complex [9,10,11]. In turn, the transcription of Wnt/β-catenin target genes, such as c-myc, cyclin D1 and Bcl-w, is activated, followed by subsequent cell proliferation that is closely related to tumorigenesis and cancer progression [12,13,14].

By contrast, in the absence of the extracellular Wnt needed for interaction with the receptors, β-catenin is degraded by a destruction complex composed of GSK-3β, adenomatous polyposis coli protein (APC), casein kinase 1α (CK1α) and Axin [15]. During the degradation process, β-catenin is initially phosphorylated by CK1α and GSK-3β, which makes it recognizable by a ubiquitin E3 ligase via β-transducin repeat-containing proteins (β-TrCP). Subsequent ubiquitination and proteasomal degradation of β-catenin occur, thereby maintaining a low intracellular level of β-catenin [16]. In this normal condition, TCF/Lef proteins are bound by repressors, such as CREB-binding protein or p300, and abnormal cell proliferation does not occur.

The Wnt/β-catenin signaling pathway is involved in the tumorigenesis of several malignancies, including colorectal cancers, non-colorectal gastrointestinal cancers, desmoid tumors, breast cancers, adrenocortical tumors, melanoma, glioblastoma multiforme, renal cell carcinoma, osteosarcoma and hematologic malignancies [5]. This pathway plays an important role in the transcriptional regulation of multiple oncogenes; therefore, it is essential for tumor occurrence and progression. Aberrant activation of this signaling pathway has been verified in various types of cancer [17]. For example, β-catenin-stabilizing mutations have been identified in colorectal cancers and have resulted in constitutive nuclear localization of β-catenin and transcription of its target genes [18].

In addition, multiple fusion transcripts, including recurrent gene fusions involving the R-spondin family members RSPO2 and RSPO3, have been identified in colon cancer cells [19]. R-spondins are proteins that enhance Wnt signaling and serve as oncogenic drivers in RSPO fusion tumors [19,20]. Hyperactivation of Wnt signaling has also been identified in hepatocellular carcinoma, which is mediated by loss of function and inactivation of the Wnt negative regulators AXIN1 and/or AXIN2 [21]. In breast and ovarian cancer cell lines, autocrine production of various Wnt ligands has been reported and has resulted in an increase in β-catenin stability [22].

Fortunately, in the last decade, many inhibitors, including biologics and small molecules, that focus on the Wnt pathway have been discovered. The biologics, which include antibodies and RNA interference molecules, mainly focus on targeting the Wnt protein itself and on the use of recombinant proteins that target extracellular modulators of this pathway [23,24,25]. By contrast, the small molecules fall into four categories based on their functional mechanism: (1) β-catenin/TCF interaction inhibitors; (2) antagonists of transcriptional co-activators, such as CBP and p300; (3) molecules binding to DVL; and (4) inhibitors based on the stabilization of Axin protein [26]. All these drugs show promising anticancer effects mediated by disrupting the signal transduction of the Wnt/β-catenin pathway.

One of the identified small molecule inhibitors of the β-catenin/TCF4 interaction is PNU-74654, a compound that prevents TCF4 from binding to β-catenin; therefore, it acts as a Wnt/β-catenin antagonist. This small molecule was discovered through virtual screening and confirmed by biophysical screening to disturb protein–protein interactions [27]. PNU-74654 competes with TCF4 for the binding site on β-catenin and its effective role as a Wnt pathway antagonist has been proven by a luciferase activity assay for TCF transactivation [27].

TCF4 is the most important transcription factor in the TCF/Lef family, as it has the ability to bind DNA and modulate the transcription of many oncogenes. Therefore, a small molecule that can block the interaction between β-catenin and TCF4 can prevent the transcription of a variety of tumor-related proteins, such as c-myc, cyclin D1, Bcl-w, MDR1, IL-8 and ZEB1 [12,13,14,28,29,30]. PNU-74654 has been shown to significantly decrease cell proliferation, increase tumor cell apoptosis, decrease nuclear β-catenin accumulation and impair CTNNB1/β-catenin expression in adrenocortical tumors [31]. PNU-74654 has also shown antitumor efficacy in breast cancers, where it causes tumor shrinkage and increased tumor cell apoptosis [32]. To our knowledge, no study has yet explored the potential for using PNU-74654 as a treatment in TC. Therefore, the aim of the current study is to evaluate the antitumor activity of PNU74654 in TC cells.

## 2. Materials and Methods

### 2.1. Cell Culture

Two human testicular teratocarcinoma cell lines, NCCIT and NTERA2, were obtained from BCRC (Bioresource Collection and Research Center, Taiwan). The cells were cultured and stored according to the suppliers’ instructions. NTERA-2 cells were maintained in high-glucose (4.5 g/L) Dulbecco’s modified Eagle medium containing 10% fetal bovine serum, 1 mM sodium pyruvate, 100 U/mL of penicillin and 100 μg/mL of streptomycin. NCCIT cells were maintained in RPMI supplemented as described above. Cells were cultivated at 37 °C in a humidified 5% CO_2_ atmosphere [33].

### 2.2. MTT Assay

The MTT assay was used to detect cytotoxicity and cell growth, as described previously [33]. Briefly, TC cells were seeded at a density of 1 × 10^4^ cells in each well of 96-well plates and incubated overnight. Then, the cells were treated with PNU-74654 (MedChemExpress, Monmouth Junction, NJ, USA) at 50–250 μM for 24 h. Then, an MTT solution (0.5 mg/mL) was added to the wells and incubated for 3 h at 37 °C. The reaction was stopped by removing the supernatant, followed by dissolving the formazan product in DMSO. The absorbance at 570 nm was measured with an ELISA reader.

### 2.3. Flow Cytometry Analysis

A flow cytometer (FACSCanto II; BD Biosciences, San Jose, CA, USA) was used to determine the cell cycle population and apoptosis percentage, as described previously [33]. An Annexin V/PI apoptosis detection kit (Elabscience Biotechnology Inc., Houston, TX, USA) was used to mark apoptotic cells. Apoptosis rates were quantified by treating the cells with PNU-74654 (0, 50 and 200 μM) for 48 h, then staining them with FITC-Annexin V and PI in the dark at room temperature, according to the manufacturer’s protocol. The fractions of cells in different phases of the cell cycle were determined by treating the cells with PNU-74654 (0, 50 and 200 μM) for 24 h. Then, the supernatant and adherent cells were harvested, washed with PBS and fixed overnight in 70% ice-cold ethanol at −20 °C. After washing, the cells were resuspended in 0.4 mL of PBS containing 4 µg/mL of propidium iodide and 0.5 of mg/mL RNase A and incubated for 30 min at 37 °C in the dark. Individual cell suspensions were analyzed by flow cytometry and cell profiles were analyzed using FlowJo software (BD Biosciences, USA).

### 2.4. Hoechst 33,342 Staining

Cells were plated in 6-well plates and incubated for 16 h. Different concentrations of PNU-74654 were added to each well and incubated for 24 h. The cells were harvested at 48 h and stained with Hoechst 33,342 (10 μg/mL) for 20 min at 37 °C. Images of the Hoechst 33,342 fluorescence were captured using a fluorescence microscope (ImageXpress PICO; excitation wavelength of 350–390 nm, emission wavelength of 420–480 nm) at 20× magnification. Apoptotic cells emitted blue fluorescence and exhibited morphological changes in the nuclei typical of apoptosis. The fluorescence staining percentage of positive cells was calculated based on cell counts made from five random visual fields, as described previously [33].

### 2.5. Human Apoptosis Array for Proteome Profiling

The Human Apoptosis Proteome Profiler™ array (R&D Systems, Minneapolis, MN, USA), which examines changes in 35 apoptosis-related proteins, was used in this study. Cells were treated with or without PNU-74654 (200 μM) for 24 h and 400 μg of total protein obtained after cell lysis was used for each array and analyzed according to the manufacturer’s instructions. Membranes were imaged by chemiluminescence and the integrated density of the spots was quantified using Image J software, as described previously [33].

### 2.6. Protein Extraction from Cells and Western Blotting

The cells were treated with PNU-74654 (0, 50 and 200 μM) for 24 h, then lysed in RIPA buffer containing a protease inhibitor cocktail (Roche Molecular Biochemicals, Basel, Switzerland). The lysate was centrifuged for 20 min at 10,400 rcf at 4 °C and the supernatant was saved. The protein concentration was measured with the Bio-Rad Protein Assay (Bio-Rad Laboratories Inc., Hercules, CA, USA). Equal amounts of protein (15 μg) per sample were boiled in sample buffer, electrophoresed on sodium dodecyl sulfate–polyacrylamide gels, then transferred onto an Immobilon^TM^-P transfer membrane (Millipore, Burlington, MA, USA). After blocking, the membranes were probed with antibodies at 4 °C for 16 h. The membranes were washed with Tris-buffered saline with 0.1% Tween 20 detergent (TTBS), then incubated for 1 h with horseradish peroxidase-conjugated secondary antibodies. The membrane was washed with TTBS buffer and the Western blots were visualized with ImmobilonTM-Western Chemiluminescent HRP Substrate (Millipore, USA). The results were displayed on an Amersham^TM^ Imager 680 (GE Healthcare, Chicago, IL, USA) and the integrated density of the spots was quantified using Image J software, as described previously [33].

### 2.7. Statistical Analysis

The statistical analyses were conducted using IBM SPSS software (version 20.0). The data are presented as the mean ± S.D. The Student’s t-test was used for continuous or discrete data analysis. All statistical tests were two-sided (SEM) and values of *p* < 0.05 were considered statistically significant (*p < 0.05; ** *p* < 0.01; *** *p* < 0.001).

## 3. Results

### 3.1. PNU-74654 Had a Cytotoxic Effect on TC Cells and Elicited Cell Death

**Figure 1:**
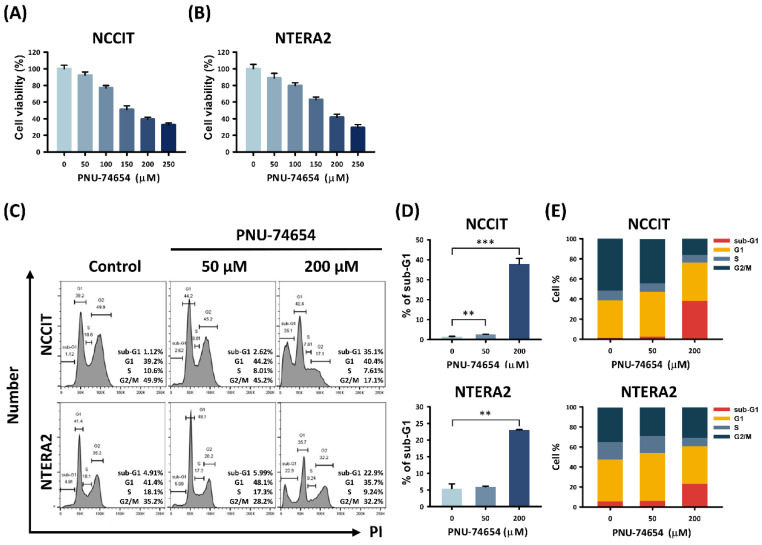
PNU-74654 caused cell death in NCCIT and NTERA2 testicular cancer cell lines. (**A**,**B**) The MTT assay determined reduced viabilities of TC cells after PNU-74654 treatment. (**C**) Flow cytometry showed increased proportions of TC cells in sub G1 phase after PNU-74654 treatment. (**D**) Columns illustrate the increased proportions of TC cells in sub G1 phase after PNU-74654 treatment. (**E**) Cell cycle distributions are shown for TC cells after PNU-74654 treatment. Data are shown as mean ± S.D (** *p* < 0.01; *** *p* < 0.001).

We confirmed the cytotoxic effect of PNU-74654 treatment on both the NCCIT and NTERA2 testicular carcinoma cell lines (Figure 1A,B). The MTT assay revealed a dose-dependent decrease in the viability of PNU-74654-treated TC cells after a 24 h exposure. Therefore, we performed a cell cycle analysis by flow cytometry to seek the causes of this cytotoxicity. Figure 1C–E shows a dose-dependent increase in the sub G1 group of TC cells after 24 h treatment with 50 and 200 μM PNU-74654 compared with the untreated control groups (NCCIT: control vs. 50 μM and 200 μM, *p* value = 0.002 and 0.001; NTERA2: control vs. 50 μM and 200 μM, *p* value = 0.564 and 0.002). The proportions of NCCIT cells in the sub G1 phase rose from 1.39% ± 0.24% (control group) to 2.58% ± 0.13% (50 μM group) and 37.93% ± 2.80% (200 μM group). The proportions of NTERA2 cells in the sub G1 phase rose from 5.41% ± 1.38% (control group) to 5.92% ± 0.24% (50 μM group) and 23.03% ± 0.12% (200 μM group). Therefore, the cytotoxicity of PNU-74654 was confirmed to cause the death of both NCCIT and NTERA2 TC cells.

### 3.2. Apoptosis Induced by PNU-74654 in TC Cells

**Figure 2:**
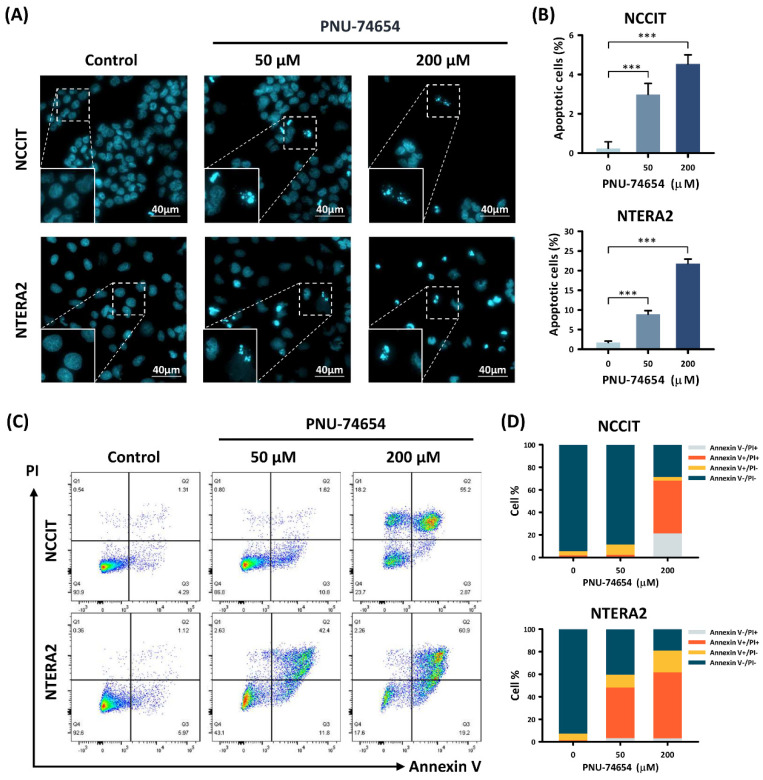
PNU-74654 caused apoptosis in TC cells. (**A**) Hoechst 33,342 staining showed apoptosis-associated morphological changes and increased numbers of apoptotic cells in TC cell lines after PNU-74654 treatment. (**B**) Bar chart shows increased proportions of apoptotic TC cells after PNU-74654 treatment. (**C**) Flow cytometry after Annexin V/PI double staining showed distributions of early apoptosis, late apoptosis and necrosis in TC cells after PNU-74654 treatment. (**D**) Columns show proportions of Annexin V^+/−^/PI^+/−^ TC cells after PNU-74654 treatment. Data are shown as mean ± S.D (*** *p* < 0.001).

We further distinguished the types of cell death occurring in PNU-74654-treated TC cells by Hoechst 33,342 staining and Annexin V/PI double staining (Figure 2). The Hoechst 33,342 staining (Figure 2A,B) revealed that the proportions of apoptotic TC cells rose dose-dependently with PNU-74654 treatment compared to the control groups (NCCIT: control vs. 50 μM and 200 μM, *p* value = 0.001 and 0.001; NTERA2: control vs. 50 μM and 200 μM, *p* value = 0.001 and 0.001). The proportions of apoptotic NCCIT cells rose from 0.24% ± 0.33% (control group) to 2.99% ± 0.56% (50 μM group) and 4.54% ± 0.46% (200 μM group). The proportion of apoptotic NTERA2 cells rose from 1.69% ± 0.41% (control group) to 8.91% ± 0.91% (50 μM group) and 21.83% ± 1.11% (200 μM group).

The Annexin V/PI double staining (Figure 2C,D) showed dose-dependent rises in the proportion of early-apoptotic (Annexin V+/PI−) and late-apoptotic (Annexin V+/PI+) TC cells with PNU-74654 treatment compared to the control groups (NCCIT: control vs. 50 μM and 200 μM, *p* value = 0.053 and 0.008; NTERA2: control vs. 50 μM and 200 μM, *p* value = 0.001 and 0.001). The proportions of apoptotic NCCIT cells rose from 4.91% ± 0.60% (control group) to 10.92% ± 2.66% (50 μM group) and 50.13% ± 7.01% (200 μM group). The proportions of apoptotic NTERA2 cells rose from 7.14% ± 0.06% (control group) to 56.47% ± 3.26% (50 μM group) and 77.87% ± 3.12% (200 μM group). In brief, the results of Hoechst 33,342 staining and Annexin V/PI double staining confirmed apoptosis as the major type of cell death in PNU-74654-treated TC cells.

### 3.3. Inhibition of TNF R1 Triggered NF-κB Anti-Apoptotic Pathway and Execution Phase of Apoptosis Contributed to PNU-74654-Induced Apoptosis in TC Cells

**Figure 3:**
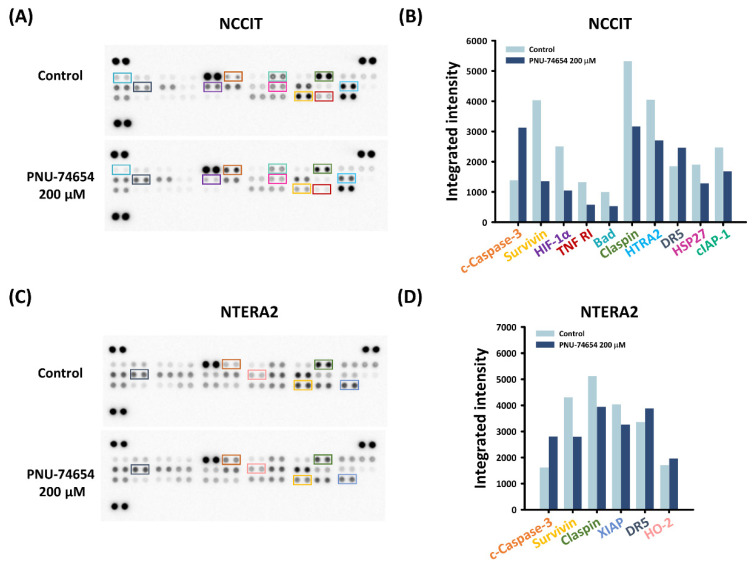
Mechanisms underlying PNU-74654-induced apoptosis in NCCIT and NTERA2 cells. (**A**,**C**) Apoptosis array testing expression of apoptosis-related proteins in TC cells after PNU-74654 treatment. (**B**,**D**) Quantitative analysis of proteins with apparent changes in integrated intensities in TC cells after PNU-74654 treatment, with cut-off >25% and <25%.

We explored the underlying mechanisms of PNU-74654-induced apoptosis in TC cells using apoptosis arrays. As shown in Figure 3A–D, after 24 h treatment with 200 μM PNU-74654, both TC cell lines showed notable upregulations of cleaved-caspase-3 and downregulations of claspin and survivin, but only NCCIT cells showed downregulations of TNF R1 and cIAP-1.

**Figure 4:**
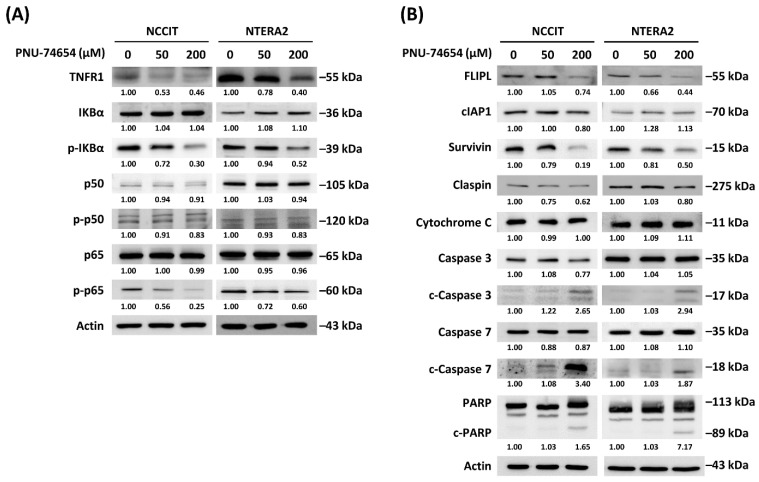
Western blots of proteins related to the execution phase of apoptosis. PNU-74654 treatment suppressed (**A**) TNFR1/IKB alpha/p65 signaling and induced (**B**) signaling pathway in NCCIT and NTERA2 TC cell lines.

We sought a deeper understanding and ascertainment by conducting Western blots to test the expressions of proteins involved in the execution phase of apoptosis and in TNFR1/IKB alpha/p65 and apoptotic signaling (Figure 4A,B). We observed inhibition of the TNFR1-triggered pathway (Figure 4A) in the form of downregulations of TNFR1, phospho-IκBα and phosphor-p65. We observed inhibition of the execution phase of apoptosis (Figure 4B) in the form of downregulations of FLIP_L_ and survivin and upregulations of cleaved-caspase-3, cleaved-caspase-7 and cleaved-PARP.

## 4. Discussion

The findings of the current study confirmed that PNU-74654 can exert a cytotoxic effect on TC cells by inducing apoptosis. When treated with PNU-74654, TC cells showed a dose-dependent decrease in viability, as determined by the MTT assay. Our cell cycle analysis revealed a significant dose-dependent increase in the sub-G1 group of TC cells treated with PNU-74654, indicating increased cell death. Our Hoechst 33,342 and Annexin V/PI double staining results further confirmed apoptosis as the major type of cell death in PNU-74654-treated TC cells. Our further analyses implicated Wnt/β-catenin signaling in this response.

Wnt/β-catenin signaling has been implicated in both germ cell tumor (GCT) progression and in treatment resistance to cisplatin-based chemotherapy [34]. Another study focusing on the aberrations of the adenomatous polyposis coli (APC) tumor suppressor in GCT arising during childhood and adolescence discovered methylation of APC and loss of heterozygosity at 5q21-22 in yolk sac tumor and teratomas [35]. To our knowledge, the present study is the first to prove a direct cytotoxic ability of a small molecule inhibitor targeting the β-catenin/TCF4 interaction in this signaling pathway.

The anti-apoptotic effect of nuclear factor kappa-light-chain-enhancer of activated B cells (NF-κB) induced by tumor necrosis factor receptor 1 (TNF R1) has been confirmed for decades [36]. We also examined the mechanisms behind PNU-74654-induced apoptosis in NCCIT and NTERA2 cells by an apoptosis array and quantitative analysis. Upregulations of cleaved-caspase-3 in TC cells, downregulations of claspin and survivin in TC cells and downregulations of TNF R1 and cIAP-1 in NCCIT cells alone were disclosed. Caspase-3 serves as a marker of programmed cell death. It is cleaved and activated following the initiation of apoptosis and its antibodies are now used as strong indicators of cell death induction [37]. By contrast, claspin acts as an adaptor or scaffold protein that facilitates the ATR-dependent activation of Chk1 [38]. Claspin undergoes cleavage and degradation by caspases and the proteasome during apoptosis [39]. Conversely, survivin is a member of the inhibitor of apoptosis (IAP) family and functions as an inhibitor of the activation of caspases and as a negative regulator of programmed cell death [40].

Our Western blot data revealed the expression of proteins involved in the execution phase of apoptosis and in the TNF R1-triggered NF-κB anti-apoptotic pathway. For the execution phase of apoptosis, we noted upregulations of cleaved-caspase-3, cleaved-caspase-7 and cleaved-PARP and downregulations of survivin and claspin. For the inhibition of the TNF R1-triggered NF-κB anti-apoptotic pathway, we observed downregulations of TNF R1, phospho-IκBα, FLIPL, phospho-p50 and phospho-p65. Therefore, we concluded that the execution phase of apoptosis and the inhibition of the TNF R1-triggered NF-κB anti-apoptotic pathway seem to be responsible for the apoptosis induced by PNU-74654 treatment of TC cells.

As already mentioned, the Wnt/β-catenin signaling pathway plays an essential role in the regulation of the transcription of a variety of oncogenes and is involved in the tumorigenesis of several cancers. Numerous medications targeting this signaling pathway have been discovered in the past decade, including the small molecule inhibitor PNU-74654 used in the current study. PNU-74654 interferes with the β-catenin/TCF4 interaction, thereby blocking the ongoing transcription of various oncogenes. The evidence in our study indicates that TC cells treated with PNU-74654 initiated a dose-dependent programmed cell death through the execution phase of apoptosis and inhibition of the TNF R1-triggered NF-κB anti-apoptotic pathway.

The Wnt signal transduction cascade mediates numerous essential cellular functions, such as proliferation, differentiation, apoptosis and cell migration, across many species [5]. Wnts are also key factors for most types of tissue stem cells in mammals [41]. In normal tissue, the β-catenin/TCF4 pathway is maintained in the “Wnt off” state, where β-catenin is phosphorylated by the destruction complex and then undergoes proteasomal degradation. This maintains a low intracellular level of β-catenin and prevents further abnormal cell proliferation. PNU-74654 blocks the interaction between β-catenin and TCF4, thereby preventing the transcription of tumor-related proteins in the affected testis or in metastatic lesions. Since the β-catenin/TCF4 pathway maintains the “Wnt off” state in normal tissue, where the intracellular β-catenin is low, the β-catenin/TCF4 interaction is not turned on. Therefore, the negative impact of PNU-74654 on the contralateral healthy testis or normal tissue can be ignored.

This result is well explained by the process of Wnt/β-catenin signaling. In a breast cancer model, PNU-74654, either alone or in combination with fluorouracil (5-FU), was shown to suppress breast cancer cell growth and to synergistically enhance the antiproliferative activity of gemcitabine [32]. The PNU-74654/5-FU combination increased the percentage of cells in the S-phase and significantly induced apoptosis in PNU-7465-treated breast cancer cells. PNU-74654 also significantly decreased cell proliferation, increased early and late apoptosis and impaired CTNNB1/β-catenin expression in an adrenocortical cancer cell model [31]. In the setting of acute myeloid leukemia (AML), higher levels of β-catenin, Ser675-phospho-β-catenin and GSK-3α were found in AML cells from intermediate- or poor-risk patients and the patients presenting with a higher activity of Wnt/β-catenin showed a shorter progression-free survival [42]. The authors of that study tested the combinatorial treatment between Wnt inhibitors and classic anti-leukemia drugs, both in vitro and in vivo and found that in vitro administration of PNU-74654, niclosamide and LiCl significantly reduced the bone marrow leukemic burden, with a synergistic effect on Ara-C, thereby improving mouse survival. These results, combined with ours, demonstrate the ability of PNU-74654 to specifically target the Wnt pathway, interfere with cell proliferation, induce apoptosis and reduce tumor cell migration. These results indicate that PNU-74654 could represent a promising therapy against several cancers.

Due to the diversity of TC types, one limitation of the current study is our use of NCCIT and NTERA2 cells only, as these two lines cannot represent all cancer cell types associated with TC. In addition, the antibody array we used to select the possible pathway leading to apoptosis has the potential flaw that we might have missed some other interesting pathway. To compensate for this limitation, we performed Western blotting to examine other interesting proteins that might be involved in apoptosis. Another limitation of our work is the lack of experimentation on animals and the lack of evaluation of treatment dosage and toxicity. In the published research works, various doses were used for different types of cancer. Doses ranging from 10 to 200 µM were used in vitro and doses ranging from 0.5 to 30 mg/kg were used in mouse models [31,32,42,43,44]. More precise evaluations will be needed before considering clinical trials.

## 5. Conclusions

PNU-74654 can induce the apoptosis of NCCIT and NTERA2 TC cells through mechanisms involving the execution phase of apoptosis and the inhibition of TNFR1/IKB alpha/p65 signaling. Along with previous studies on the antitumor effects of PNU-74654, the present findings may provide preliminary evidence to support further experiments on the use of PNU-74654 as a potential new treatment strategy for patients with TC.

## Data Availability

All data analyzed are included in this article is available upon request.
